# Long-term cardiovascular disorders in the STOX1 mouse model of preeclampsia

**DOI:** 10.1038/s41598-019-48427-3

**Published:** 2019-08-15

**Authors:** Francisco Miralles, Hélène Collinot, Yasmine Boumerdassi, Aurélien Ducat, Angéline Duché, Gilles Renault, Carmen Marchiol, Isabelle Lagoutte, Céline Bertholle, Muriel Andrieu, Sébastien Jacques, Céline Méhats, Daniel Vaiman

**Affiliations:** 1 Institut Cochin, U1016 INSERM - UMR8104, CNRS – Université Paris Descartes, Team “From Gametes To Birth”, 24 rue du Faubourg St Jacques, 75014 Paris, France; 2 Institut Cochin, U1016 INSERM - UMR8104, CNRS – Université Paris Descartes, Genom’IC Platform, Bâtiment Gustave Roussy, 27 rue du faubourg Saint Jacques, 75014 Paris, France; 3 Institut Cochin, U1016 INSERM - UMR8104, CNRS – Université Paris Descartes, PIV Platform, 22 rue Méchain, 75014 Paris, France; 4 Institut Cochin, U1016 INSERM - UMR8104, CNRS – Université Paris Descartes, CYBIO Platform, 27 rue du Faubourg Saint Jacques, 75014 Paris, France

**Keywords:** Cardiac hypertrophy, Pre-eclampsia

## Abstract

Adverse long-term cardiovascular (CV) consequences of PE are well established in women. However, the mechanism responsible for that risk remains unknown. Here, we mated wild-type female mice of the FVB/N strain to STOX1A-overexpressing mice to mimic severe PE and investigated the long-term consequences on the maternal cardiovascular system. Ultrasonography parameters were analyzed in mice before pregnancy and at 3 and 6 months post-pregnancy. At 6 months post-pregnancy, cardiac stress test induced by dobutamine injection revealed an abnormal ultrasonography Doppler profile in mice with previous PE. Eight months post-pregnancy, the heart, endothelial cells (ECs) and plasma of females were analyzed and compared to controls. The heart of mice with PE showed left-ventricular hypertrophy associated with altered histology (fibrosis). Transcriptomic analysis revealed the deregulation of 1149 genes in purified ECs and of 165 genes in the hearts, many being involved in heart hypertrophy. In ECs, the upregulated genes were associated with inflammation and cellular stress. Systems biology analysis identified interleukin 6 (IL-6) as a hub gene connecting these pathways. Plasma profiling of 33 cytokines showed that, 8 of them (Cxcl13, Cxcl16, Cxcl11, IL-16, IL-10, IL-2, IL-4 and Ccl1) allowed to discriminate mice with previous PE from controls. Thus, PE triggers female long-term CV consequences on the STOX1 mouse model.

## Introduction

Preeclampsia (PE), a major hypertensive disease of pregnancy, is an important risk factor for cardiovascular disease (CVD) in women^1^. PE symptoms appear from mid-pregnancy and induce maternal hypertension and proteinuria. PE affects ~5% of pregnancies and remains a major cause of maternal morbidity and mortality^2^. In PE, defective vascularization of the placenta causes the production and release of anti-angiogenic factors. These factors affect the maternal cardiovascular system (CVS) triggering a systemic endothelial activation responsible for hypertension and kidney dysfunction^3^.

The symptoms of PE disappear after delivery, but numerous epidemiological studies have revealed long-term consequences for maternal health. A recent systematic analysis associated PE conditions to injury to the maternal CVS^1,4,5^. About 20% of women with PE show hypertension or microalbuminuria within 5 years after delivery, as compared with only 2% of women with normal pregnancies^6,7^. In addition, the long-term risk of cardiovascular and cerebrovascular disease is doubled in women with PE, as compared with women of the same age. This risk is even higher for women with recurrent preeclamptic pregnancies^8^. For women with early PE (before 34 weeks’ amenorrhea) or PE associated with intrauterine growth retardation, the risk of death from CVD is 4 to 8 times higher than those with normal pregnancies^9^. A recent review detailed the long-term consequences of PE in women in terms of increased risk of CVD, kidney disease or stroke^10^.

The mechanisms responsible for these risks after PE are not known, although microvascular dysfunction induced by PE has been linked to maternal coronary heart disease 15 to 25 years after PE^11^. The long-term increase in CVD in women with PE may result from shared risk factors, subtle vascular damage, or persistent endothelial dysfunction caused by the disease and playing a synergistic harmful role with the aging of the vascular system. None of these (not mutually exclusive) assumptions is currently favored.

Studies on women are essentially limited to epidemiological analyses and have not examined relevant tissues by molecular biology methods. A recurrent question is whether the increased risk of CVD after PE is directly induced by the gestational disease or results from a specific genetic background in the patient that causes the PE and later the CVD and other manifestations. Several studies have used animal models of PE to evaluate the long-term consequences on the mother at various times post-pregnancy and on specific outcomes, such as blood pressure, vascular reactivity, proteome, abnormal heart structure, vascular reactivity, aortic function, brain inflammation^12–15^.

Our team developed a mouse model of severe and early PE by overexpressing the storkhead box 1A (STOX1A) isoform of STOX1 protein, a transcription factor involved in the development of PE^16^. Wild-type females with at least one placenta expressing the transgene develop a syndrome like human PE (Supplementary Fig. 1). Blood pressure increases from 116 to >160 mmHg^17^. Animals also show increased proteinuria, kidney fibrosis, heart hypertrophy and endothelial transcriptomic alterations^17,18^. These symptoms disappear after delivery. Hence, STOX1 mice are particularly suited to study the effects of severe early PE on the maternal CVS. In the present study, mice with PE and those with normotensive gestation were maintained for 8 months (more than one quarter of an average lifespan) and their cardiovascular function was reanalyzed. We detected 8 months post-PE, left-ventricular (LV) hypertrophy, fibrosis, altered cardiac function upon dobutamine stress, modified endothelial and cardiac transcriptome and mildly altered cytokine profile.

## Methods

### Animals

Mice were raised as described^19^, and experiments were approved by the Animal Care Committee of the Paris Descartes University (agreement no. 02731.02). The outline of the experiment is shown in Supplementary Fig. 1. All females used in the study were wild-type (WT) females of the FVB/N strain. After their pregnancy that occurred from age 8 to 12 weeks, they were maintained for 8 months in cages. All methods were performed in accordance with the relevant guidelines and regulations (APAFIS #18-014).

Pregnancy was induced in half of the females by mating with males overexpressing the STOX1A transcription factor (TgSTOX13 from the FVB/N background) and others were mated with FVB/N WT males. After pregnancy, the mice were randomized in two large cages (5 animals per cage) and maintained for the duration of the experiment. Blood pressure was analyzed at 3 and 6 months post-pregnancy by tail-cuff plethysmography as described^17^.

### Experimental procedures

The procedures for ultrasonography (US), histology, EC purification, transcriptomics and bioinformatics analyses, circulating cytokine quantifications and statistics are in Supplementary Methods.

## Results

### Heart structure and function do not recover in mice with previous PE versus controls

**Figure 1:**
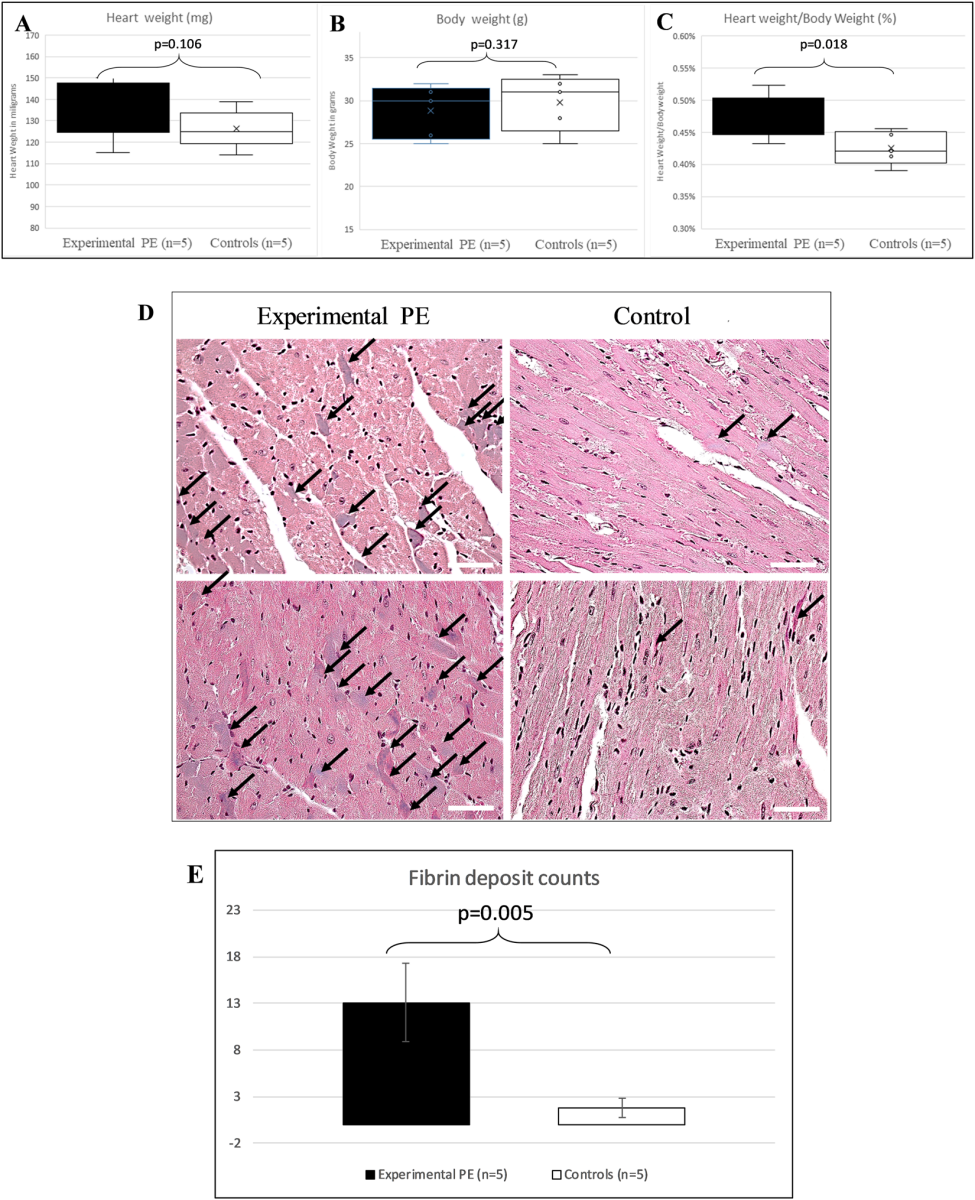
(**A**–**C**) Heart and body weight of mice 8 months after preeclampsia (experimental PE) or a normotensive pregnancy (controls). (**A**) Heart weight of animals was measured and plotted as a whisker plot. (**B**) Body weight of mice. (**C**) Relative heart weight (heart was divided by mouse total weight and the percentage is plotted). (**D**) Heart histology in mice with preeclamptic pregnancy (experimental PE) or normotensive pregnancy (control), with fibrin deposits stained in purple as revealed by Masson trichrome staining; black arrows show fibrin deposits. (**E**) Fibrin deposits. Data are mean ± SD. P value calculated by Student *t* test.

Eight months after pregnancy (preeclamptic or normotensive) the hearts of mice were weighed and normalized against whole-body mouse weights (Fig. 1A). The relative heart weight ranged from 0.39% to 0.52%. The mean relative weight was 0.473% and 0.425% in mice with previous PE versus control mice, representing a 10.1% increase (p = 0.018), similar to what we observed in previous studies at the end of pregnancy^18,20–22^. We also analyzed blood pressure of these ageing mice and found systolic blood pressure (BP) increased 1.5 mm Hg and diastolic BP increased 3.5 mm Hg in mice with previous PE versus controls, although not significant (p > 0.05, data not shown). In Fig. 1B, Masson histology staining of the mouse heart shows remnant fibrosis marks in several cardiomyocytes, that appeared blue-purple. The two groups differed in mean number of fibrosis deposits (13.00 ± 2.35 vs 1.75 ± 2.06 for experimental PE and control mice; p = 0.005, Fig. 1E).

We collected 59 ultrasonography (US) parameters for analyzing heart function of the 2 mouse groups as described^19^, before pregnancy, at 3 and 6 months post-pregnancy. At 6-months the US analysis was conducted with and without dobutamine injection (Supplementary Table 1). This drug increases cardiac output, heart rate and myocardial oxygen demand and is generally used to mimic cardiac stress^23^. We first showed an effect of time of measurement (before pregnancy, 3 and 6 months) for 9 parameters (aortic flow, interventricular septum thickness, aortic valve velocity-time integral and pressure, pulmonary artery mean and peak gradient pressure) and an interaction effect according to the occurrence of PE during pregnancy, for 4 parameters, 3 of which are linked to pulmonary artery flow and pressure measures.

**Figure 2:**
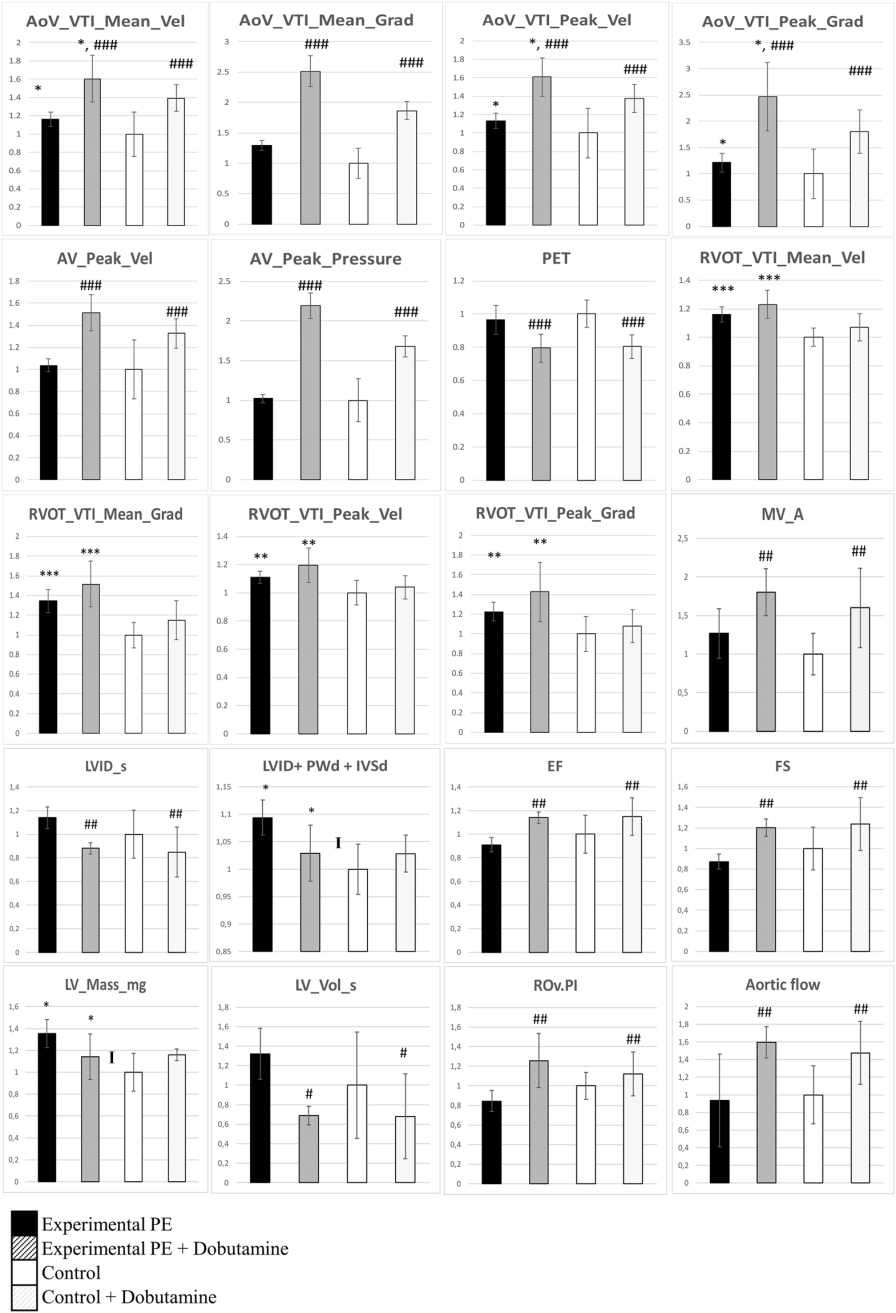
Multiple ultrasonography (US) parameters measured in the two groups of mice using a VisualSonic VEVO2100, with mice lightly anesthetized under isofluorane, 6 months after pregnancy. Mice were treated with dobutamine (mimicking an effort stress [see Material and Methods]) or not. Parameters were evaluated by Doppler US and direct measures for geometrical parameters of the heart. Graphs represent the normalized levels for each group, the control group without dobutamine was the reference. Data are mean±SD analyzed by two-way ANOVA followed by Student-Neumann-Keuls post-hoc tests. The hash (#) and asterisk (*) symbols mark the dobutamine effect and the genotype effect (pathological pregnancy), respectively, with *^,#^ for p < 0.05, **^,##^ for p < 0.01 and ***^,###^ for p < 0.001. The “I” marks an interaction effect between the two parameters measured. In the graph, among 59 parameters measured, 20 with significant values are presented. The significance of the US abbreviations are given in Supplemental Table 1. In the figure, AoV_VTI_Mean_Vel, mean ascending aorta peak velocity; AoV_VTI_Mean_Grad, mean ascending aorta peak gradient; AoV_VTI_Peak_Vel, ascending aorta mean velocity; AoV_VTI_Peak_Grad, ascending aorta mean pressure gradient; AV_Peak_Vel, ascending aorta peak velocity; AV_Peak_Pressure, ascending aorta pressure gradient; PET, pulmonary artery ejection time; RVOT_VTI_Mean_Vel, pulmonary artery mean velocity; RVOT_VTI_Mean_Grad, pulmonary artery mean velocity gradient; RVOT_VTI_Peak_Vel, pulmonary artery peak velocity; RVOT_VTI_Peak_Grad, pulmonary artery peak velocity gradient; MV_A, mitral valve a wave peak velocity; LVID_s, left ventricle internal diameter, systole; LVID + PWd + IVSd, calculation of the sum of LV internal diameter, LV posterior wall thickness and interventricular septum thickness; EF, ejection fraction (extrapolated from M mode); FS, fractional shortening; LV_Mass_mg, LV mass; LV_Vol_s, telesystolic LV volume; ROv.PI, pulsatility index; Aortic flow, flow rate in the aorta.

We then analyzed the effect of dobutamine at 6 months (Fig. 2 and Supplementary Table 1). Overall, 24 of 59 parameters were modified by dobutamine treatment, which is consistent with increased cardiovascular stress, as expected. Ten significant parameters were associated with the conditions of the previous pregnancy (experimental PE vs Control). We found an interaction effect between the 2 groups for 3 parameters. At 6 months post-pregnancy, as compared with controls, experimental PE mice showed increased ascending aorta velocity and pressure gradients (Fig. 2), increased pulmonary artery velocity and gradient-associated parameters (for pulmonary artery), increased US-estimated heart weight combined with an abnormal response to dobutamine as shown in the parameter LVID + PWd + IVSd and LV mass, for which an interaction effect (“I” in Fig. 2) was detected. This observation implies a defective heart adaptation to stress in mice with a preeclamptic pregnancy.

In summary, the cardiac hypertrophy induced by PE that we described in a previous study^18^ was not corrected 6 or 8 months later in mice. When 3 stresses are combined (aging + previous preeclamptic pregnancy + dobutamine treatment), the functional capacity of the heart is reduced.

### Transcriptional alterations of hearts at 6 months after a preeclamptic pregnancy

To better understand the long-term cardiac phenotype, we analyzed global changes in cardiac gene expression 8 months after PE in parallel with the endothelial cells (accession number in EBI-EMBL E-MTAB-7357 and E-MTAB-7358). We identified 165 differentially expressed genes (DEGs) with a fold change more than 1.5-fold (p ≤ 0.05). Of these, 105 were upregulated and 60 downregulated. Enrichment analysis was performed to gain insight into the biological role of the differentially expressed genes (Table 1). Among the upregulated genes the most over-represented Gene Ontology terms (GO) in the biological process category were related to “protein localization in the endoplasmic reticulum”, “translation initiation”, “muscle cell migration” and “fatty acid metabolism”. In the category of GO cellular component, the most enriched terms were related to “ribosome proteins”, and “respiratory chain complex”. Pathways analysis (using KEGG, Reactome and Wikipathways databases) confirmed that the upregulated DEGs are associated mostly with the ribosome and oxidative phosphorylation. Downregulated genes showed significant enrichements, for the “NOD-signalling”, and “interferon gamma signalling” pathways.

**Table 1:** Summary of gene pathways statistically enriched in the hearts 8 months post-pregnancy.

Gene Set	Description	Size	Expect	Ratio	P Value	FDR
**Up**-**regulated genes**						
GO: Biological Process						
GO:0070972	protein localization to endoplasmic reticulum	137	0.619	20.97	3.37e-14	2.86e-11
GO:0006413	translational initiation	192	0.868	14.96	2.67e-12	1.13e-9
GO:0014812	muscle cell migration	79	0.357	13.98	0	0.0034
GO:0006631	fatty acid metabolic process	357	1.615	4.333	0.001	0.095
GO:Celular Components						
GO:0005840	ribosome	229	1.340	13.42	4.44e-16	7.63e-14
GO:0098798	mitochondrial protein complex	266	1.557	5.78	0	0.001
GO:0070469	respiratory chain	100	0.585	10.25	0	0.001
GO:0030055	cell-substrate junction	411	2.405	3.74	0.0006	0.016
Pathways						
hsa03010 (K)	Ribosome	153	1.003	15.94	8.88e-16	2.89e-13
hsa00190 (K)	Oxidative phosphorylation	133	0.872	6.87	0.00020	0.019
hsa00980 (K)	Metabolism of xenobiotics by cytochrome P450	76	0.498	8.02	0.0015	0.069
hsa05418 (K)	Fluid shear stress and atherosclerosis	139	0.911	5.48	0.0021	0.074
WP477 (W)	Cytoplasmic Ribosomal Proteins	91	0.685	18.97	5.20e-14	2.28e-11
WP623 (W)	Oxidative phosphorylation	62	0.466	8.57	0.0011	0.12
**Down**-**regulated genes**						
GO:Biological Process						
GO:1901652	response to peptide	487	1.24	7.20	0.0000029	0.0025
GO:0006631	fatty acid metabolic process	357	0.91	7.64	0	0.0126
GO:0071887	leukocyte apoptotic process	107	0.27	14.56	0.00020	0.0334
GO:0022407	regulation of cell-cell adhesion	383	0.98	6.10	0.00040	0.0421
GO:0048545	response to steroid hormone	388	0.99	6.02	0.00040	0.0421
Pathways						
WP619 (W)	Type II interferon signaling (IFNG)	37	0.14	40.50	4.6234e-9	0.0000020
R-HSA-877300 (R)	Interferon gamma signaling	92	0.27	18.50	0.0000064	0.011
hsa04621 (K)	NOD-like receptor signaling pathway	168	0.67	8.89	0	0.012
hsa04620 (K)	Toll-like receptor signaling pathway	104	0.41	9.57	0.00070	0.06

**Figure 3:**
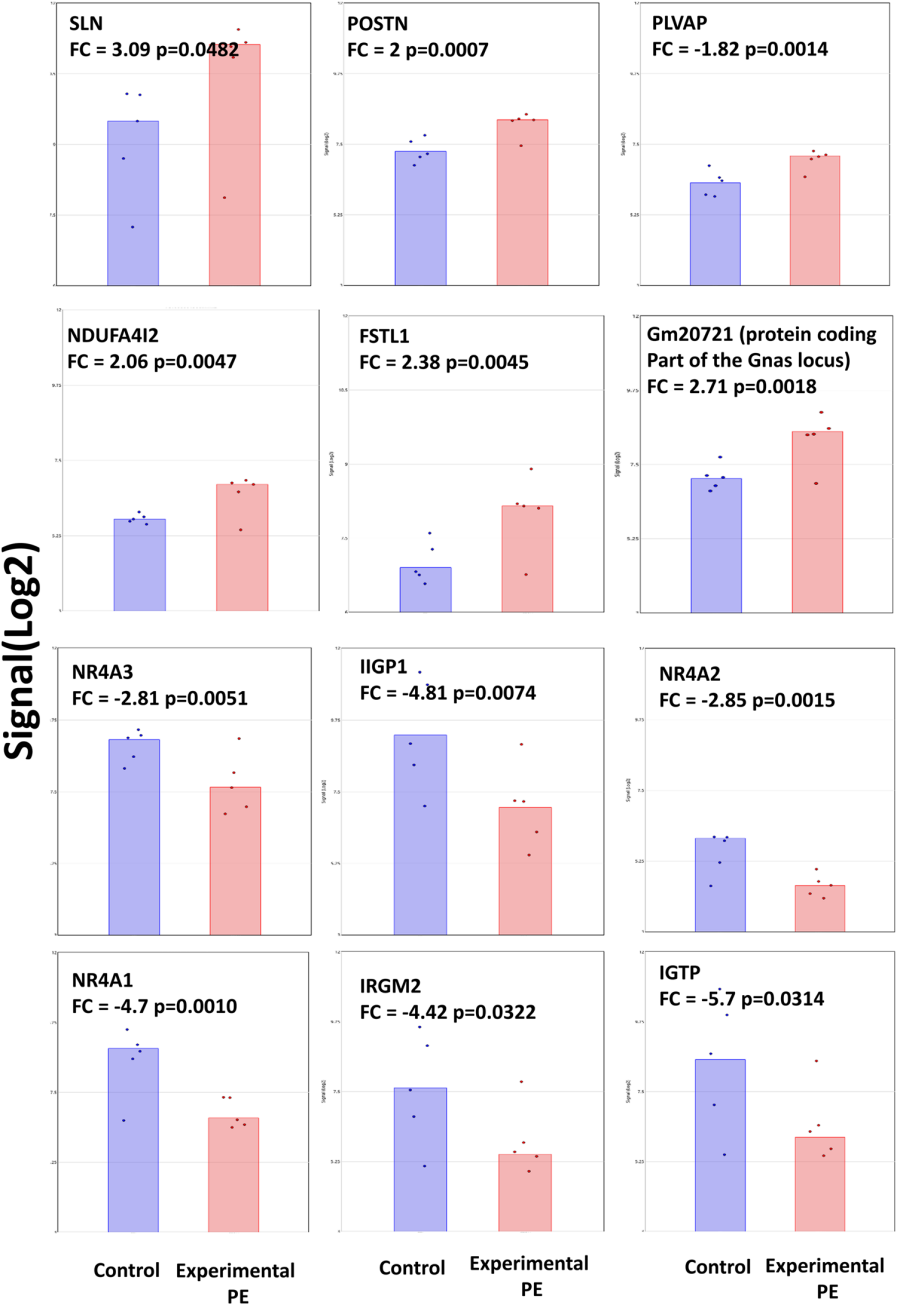
Deregulation of genes in the heart of mice having had a preeclamptic pregnancy 8 months earlier. The 6 most induced genes are presented in the upper part, the 6 most down-regulated genes are presented in the down part of the figure. In are the expression in control hearts, in red expression levels from hearts of mice having had a preeclamptic pregnancy. FC stands for fold-change, and the signals are expressed in Log2(fluorescence).

Several of the most upregulated genes in the hearts of the females who had previously experienced PE are related to cardiac hypertrophy. These include: *SLN* (Sarcolipin, FC = 3), *Fstl1* (follistatin-like 1, FC = 2.38), *Ndufa4l2* (NADH dehydrogenase (ubiquinone) 1 alpha subcomplex, 4-like 2, FC = 2), *Postn* (Periostin, osteoblast specific factor, FC = 2) and *Plvap* (plasmalemma vesicle associated protein, FC = 1.82). Amongst these up-regulated genes, we also found *Gm20721*, a mouse protein coding locus (318 amino-acids), sharing many exons with several isoforms of Gnas, an imprinted gene characterized by a very complex splicing and expression profile. The most strongly down-regulated genes have been also associated with cardiac hypertrophy including *Igtp* (interferon gamma induced GTPase, FC = −5.7), *Ligp1*(interferon inducible GTPase 1, FC = −4.8), *Irgm2* (immunity-related GTPase family M member 2, FC = −4.42), and the three members of the nuclear receptors NR4A family: *Nr4a1* (FC = −4.7), *Nr4a2* (FC = −2.85) and *Nr4a3* (FC = −2.81). The expressions of these genes are summarized in Fig. 3.

### Massive transcriptional alterations of ECs at 6 months after a preeclamptic pregnancy

**Figure 4:**
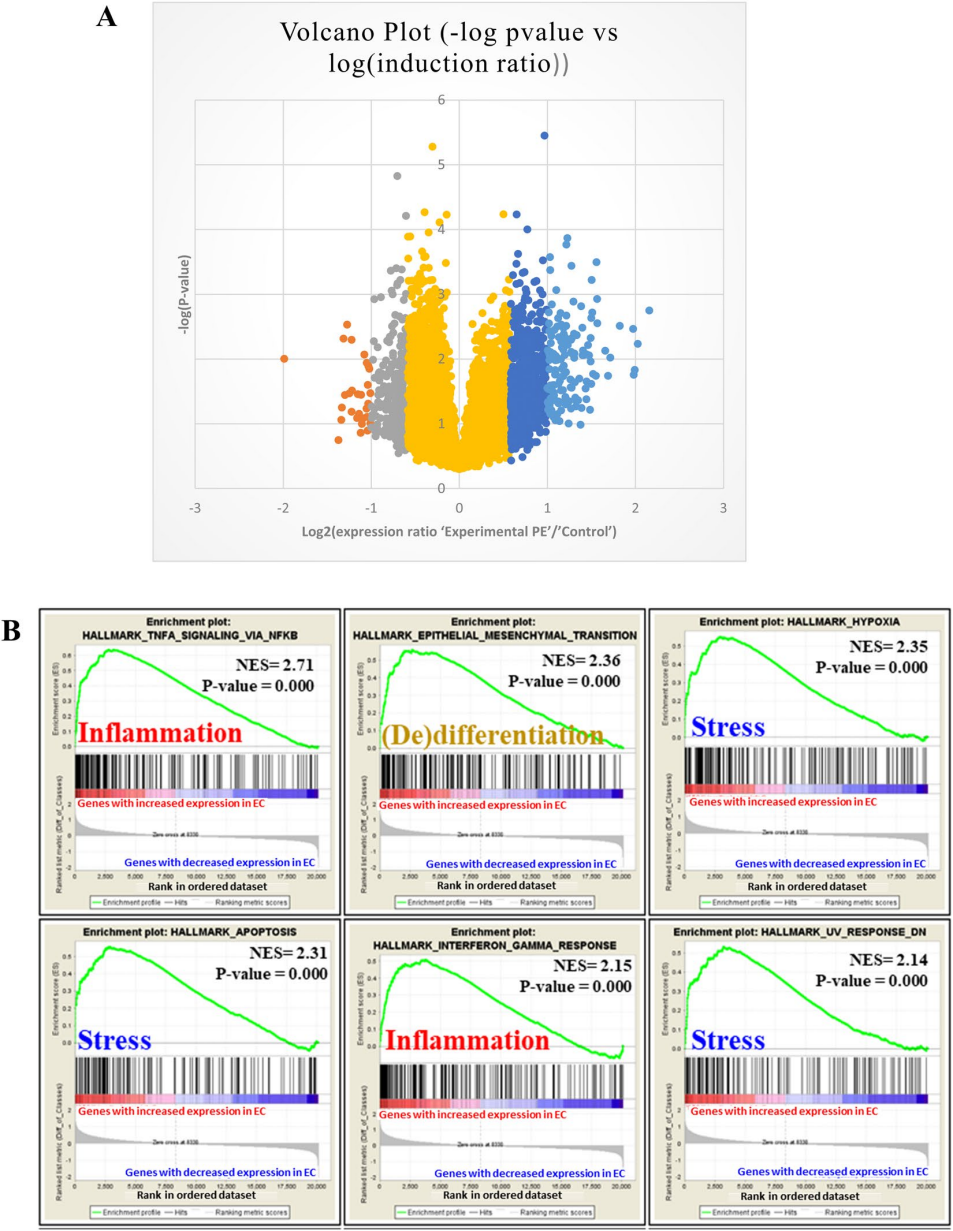
(**A**) Volcano plot of the genes differentially expressed in purified endothelial cells. Yellow represents 18997 genes that were not modified; grey, the 297 genes with expression decreased from 1.5 to 2-fold; orange, the 30 genes with expression decreased more than 2-fold; dark blue, the 682 genes with expression increased from 1.5 to 2-fold; light blue, the 140 genes with expression induced more than 2-fold. (**B**) Clustering by Gene Set Enrichment Analysis of the 20146 genes analyzed in the microarray analysis. The figure represents the 6 most significant pathways; the complete list (with the default threshold fixed at false discovery rate <0.25) is in Supplementary Fig. 2. Pathways of stress and inflammation are considerably enriched in upregulated genes, for a marked pro-inflammatory status of endothelial cells in mice with preeclampsia 8 months earlier versus mice with a normotensive pregnancy.

ECs were purified on cd31 affinity columns as described^18^ and the RNA was hybridized on ClariomS mouse microarrays (data submitted to EBI-EMBL with the accession number E-MTAB-7358). Among 20146 genes, 170 were modified by >2-fold change (30 downregulated and 140 upregulated), and 979 were modified more than 1.5-fold (297 downregulated and 682 upregulated) (Fig. 4A); 3073 genes showed differential expression at p < 0.05. Combining the 1.5-fold change and p < 0.05 thresholds, 713 genes were modified (165 downregulated and 548 upregulated). The complete dataset was analyzed by using Gene Set Enrichment Analysis (GSEA, http://software.broadinstitute.org/gsea/index.jsp), especially comparing the transcriptome data vis a vis the “Hallmarks” database, which includes 50 low-redundancy gene clusters of genes involved in important biological functions. Upregulated genes were easily clustered (40 clusters with FDR > 0.25), but we found only 3 significant clusters of downregulated genes (Supplementary Fig. 2).

**Figure 5:**
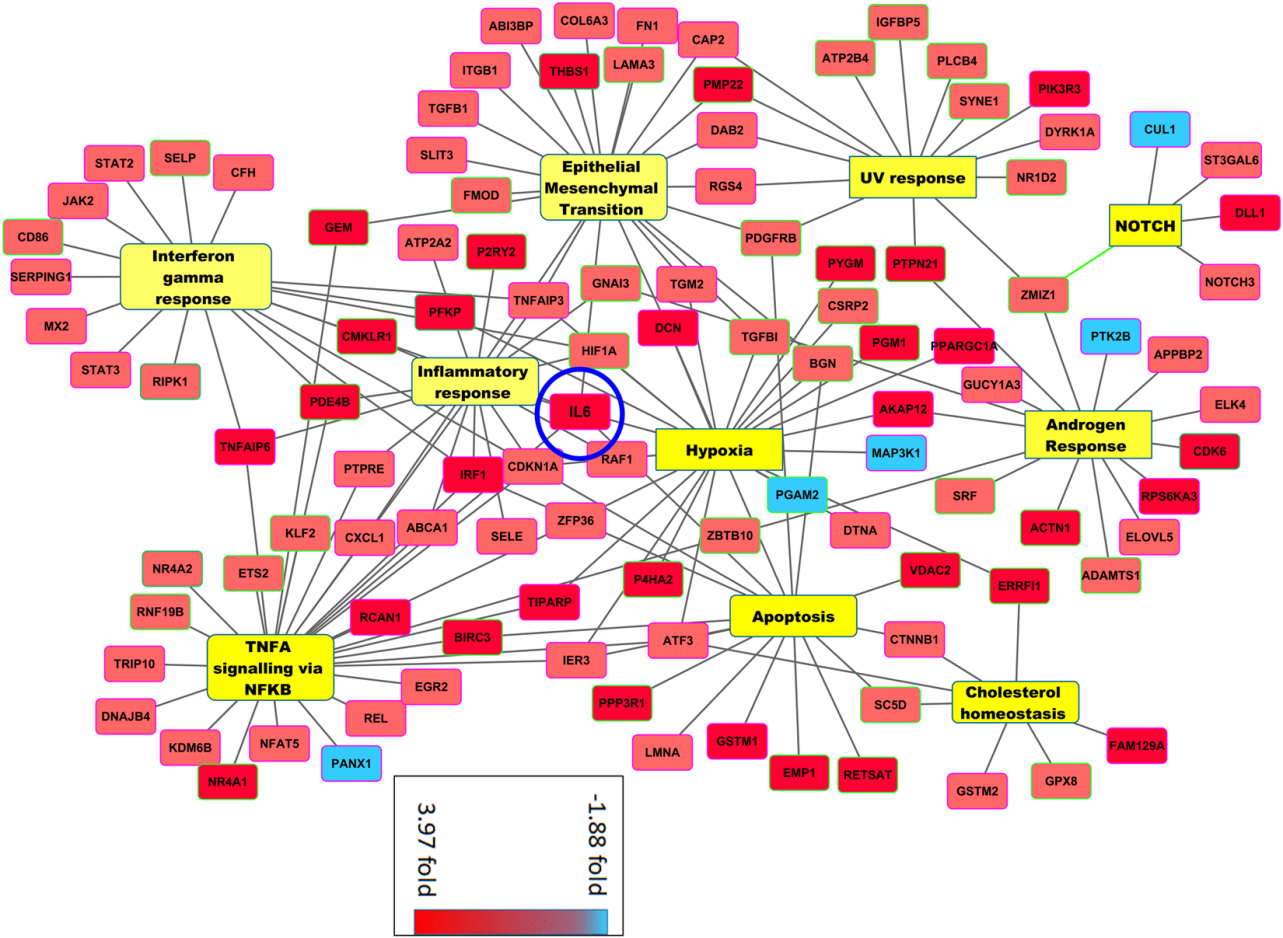
Cytoscape analysis of genes deregulated in endothelial cells. In this clustering, downregulated genes (in blue) are rare as compared with upregulated genes (in red). Il-6 gene (induced almost 4-fold) was revealed as a hub by network analysis, and thus occupies a central position in the center. It is part of several different hallmarks identified as enriched by GSEA (onflammatory response, interferon γ response, TNF-α signaling via NFκB, apoptosis and hypoxia), which suggests that in the studied context, IL-6 actually plays the role of a hub gene.

The 5 genes with the most increased expression in the upregulated group were *Rab8b*, *Rpl7l1*, *Il*-6, *Rasa1* and *Retsat* (induced 4.46-, 4.08-, 3.98-, 3.95- and 3.93-fold, respectively, with p = 0.0018, 0.0058, 0.0148, 0.0175, and 0.0034, respectively). *Il*-6 encodes a cytokine involved in various inflammatory processes. The 6 most significant GSEA clusters are presented in Fig. 4B. Hence, the alterations in the endothelium corresponded to a concerted alteration of enhanced gene expression. The genes identified were essentially related to inflammation, cell differentiation, cell stress response (Fig. 4B and Supplementary Fig. 2). In the pathways related to stress, we found “hypoxia”, “apoptosis”, and “UV response”, with highly significant normalized enrichment scores (NESs, ranging from 1.89 to 2.35 – this score measures the enrichment of genes in each pathway corrected by the number of genes included in the pathway) with FWER p values < 0.001. Inflammation pathways were deregulated as well: “TNFα signaling via NFκB”, “interferon γ response”, “inflammatory response”, and “IL-2 STAT5 signaling”, with NESs ranging from 1.90 to 2.71, and FWER p-values ≤ 0.001. To show this clusters and their functional interactions at the gene level, we present a Cytoscape map in Fig. 5. Within the network of deregulated genes composing the clusters identified by GSEA, IL-6 appears as a hub gene.

### Altered cytokine profiles in mice with a previous preeclamptic pregnancy

**Figure 6:**
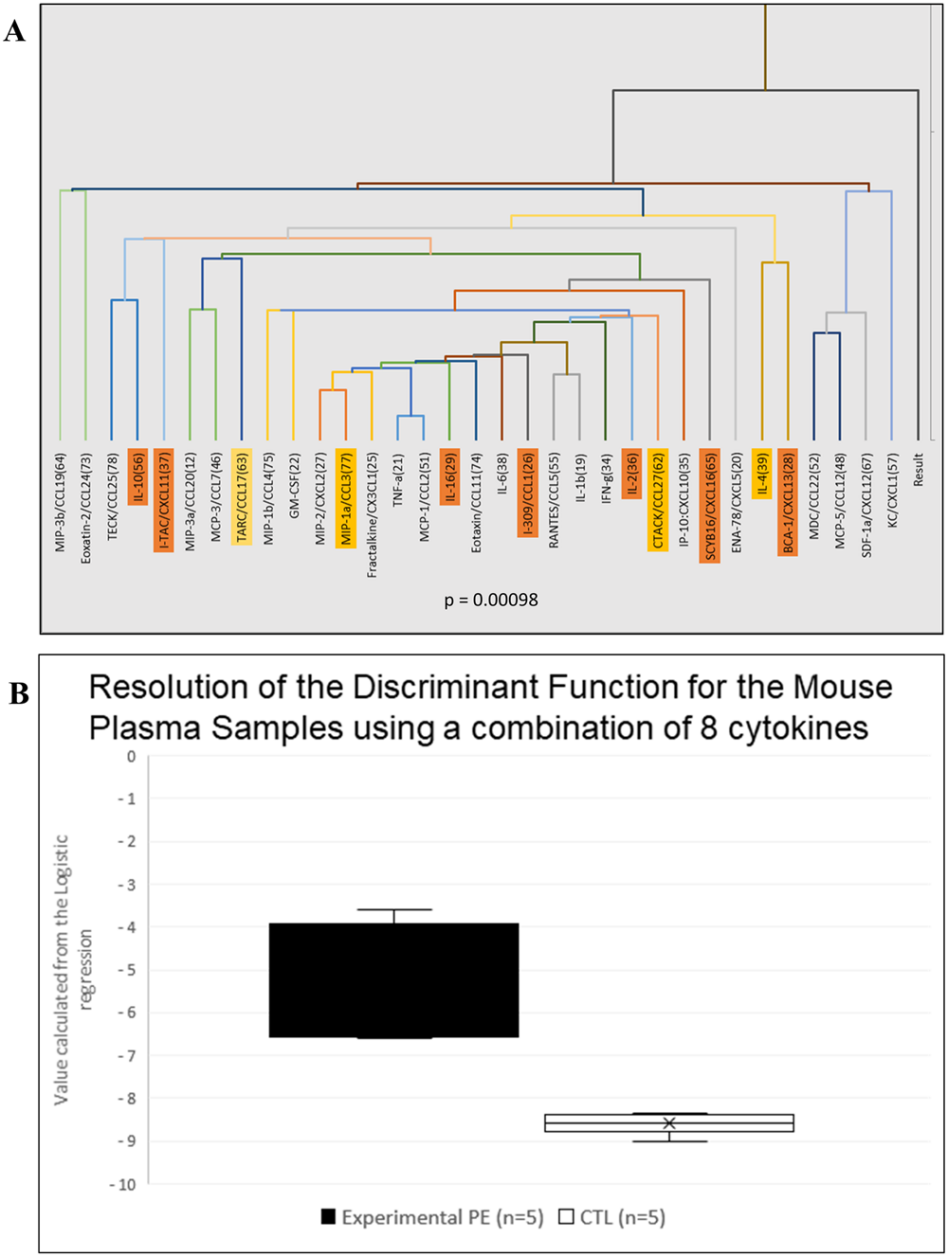
(**A**) 33 cytokines were analyzed by Luminex technology with the BioPlex kit from BioRad. A correlation analysis was performed to identify cytokines with consistent expression (see text); this approach allows for identifying several markers that behave similarly. Orange are the markers with the largest discriminatory power (see text). (**B**) 8 cytokines selected for discriminant analysis allowing for generating a linear equation whose coefficients were estimated and unambiguously classifying the 10 mice from the estimated concentrations of these 8 cytokines: Cxcl13, Cxcl16, Cxcl11, Il-16, Il-10, Il-2, Il-4 and Ccl1. The ordinates are the calculated values of the mathematical equation described in the text. The concentration of every cytokine has a multiplicative coefficient, thereby allowing to assign a “cytokine coefficient” to each mouse. Statistics were based on Student *t* test.

By using the Bioplex Pro kit from BioRad, we analyzed 33 cytokines in plasma of 5 control and 5 previous preeclamptic mice. The profile of the cytokines is presented in Fig. 6A analyzing the possible correlation between the responses. We found a highly correlated response for TNFα and CCL2 (r = 0.924), IL-1β and CCL5 (Rantes, r = 0.848), CXCL2 and CCL3 (r = 0.811).

Individually, no single marker significantly differed between the 2 mouse groups. Using a discriminant analysis method (DA), we could define a discriminant equation with the 8 best markers (lowest p-values): Cxcl13, Cxcl16, Cxcl11, Il-16, Il-10, Il-2, Il-4 and Ccl1 (normalized plasma levels are in Supplementary Fig. 3). The calculation process is presented in Supplementary Table 2, with the individual values obtained from each animal. Applying the DA equation to the concentrations of the different cytokines, the mean values for the discriminating factor were −5.39 ± 1.39 and −8.59 ± 0.25 (p = 0.00098) for the experimental PE and control groups, respectively (Fig. 6B), which suggests that the cytokine profiles allow for unambiguously discriminating the 2 groups. To connect these findings with the EC expression profiles, we observed that the altered expression of 7 of the 8 selected markers was consistent in the endothelium (except for Il-16, whose expression was decreased in ECs and increased in plasma [Supplementary Fig. 4]). Overall, the altered endothelial expression of genes encoding the cytokines in the BioPlex kit ranged from −0.17% (for Ccl1 and Cxcl11) to 397% for IL-6. Correlation was mainly found with markers that were downregulated in PE. Unexpectedly, the markers induced at the highest level (e.g., TNFα, Cxcl2, Ccl11, Ccl19, Cxcl1 and in particular Il-6) in previously preeclamptic mice were not influential in differentiating the 2 groups of mice.

## Discussion

Epidemiological studies suggest that PE represents a risk factor for CVD in the long-term after pregnancy^10^. In this study, using the STOX1 model of PE, we demonstrate that PE can produce long-term effects on the health of the maternal CVS by itself (independent of any predisposition condition). The STOX1 transcription factor is the first gene identified by positional cloning in preeclamptic human families^16^. Previous results indicated that the STOX1 mode of action is based on altered cytotrophoblast function^24^, associated with marked perturbations of the balance between oxidative and nitrosative stresses in the placenta^25^. In a previous study, we observed in this model that, at delivery, the females who experienced PE presented heart hypertrophy heart histological abnormalities (fibrosis), kidney glomerulitis as well as a modified transcriptome profile of the endothelial cells (ECs)^18^. The analysis of other models of preeclampsia in rodents could end up with additional knowledge that would be useful for understanding PE in humans^26^.

Here, we found that 8 months after pregnancy, females who experienced PE showed persistent heart LV hypertrophy, altered cardiovascular function after dobutamine stress and modified cytokine profile in plasma. In addition, in these animals, the transcriptome of purified ECs showed 1149 deregulated transcripts, while the heart presented with 165 deregulated transcripts. Bioinformatics indicated that the EC gene expression profile is reminiscent of EC dysfunction (pro-inflammatory state).

Generally, LV hypertrophy is considered to be a cardiac response to chronic ventricular pressure overload due to hypertension. Reversible LV hypertrophy without fibrosis occurs during normal pregnancy^27^ but this is not the case in PE for a substantial number of women. Thus, our results are consistent with studies reporting significant cardiac remodeling in women who had PE, in particular severe PE^28,29^. This remodeling persists often after delivery and has been associated with increased risk of heart failure^30^.

In addition to changes in size, and cardiac function, we observe, 8-months post-partum, significant changes in gene expression in the hearts of the females who had preeclamptic pregnancies. Among the most significantly DEGs we detected upregulation of Sln, Fstl1, Postn, Plvap, Gm20721, and down regulation of IFN‐γ GTPAses and nuclear receptors of the NR4A family. All these genes have been associated with CH^31–40^. In summary, the transcriptomic analysis of the hearts (8-months post pregnancy) of the mice females who experienced PE shows a significant number of DEGs compared to the females who had non-pathological pregnancies. These DEGs belong to biological pathways who have been associated with cardiac hypertrophy or cardiac dysfunction. The most upregulated DEGs have been associated with the promotion of CH (Sln, Postn, Plvap, and genes encoding ribosomal proteins). However, we observe also upregulation of FSTL1 which according to recent studies will rather have an attenuating effect on CH. Gm20721 is a protein encoded from the complex Gnas locus. Interestingly, this locus has often been associated to heart function/dysfunction in humans and mice. Several variants of GNAS have been associated to heart function^41^.

On the other hand, the most significantly dowregulated DEGs are involved in mechanisms exerting protective roles against CH (IFN-y and NR4A signaling),^38,42,43^). Importantly, we do not observe any significant changes in genes encoding cytokines, or extracellular matrix components. Therefore, it is difficult to infer from this transcriptomic data if those changes in gene expression are merely adaptive modifications to preserve cardiac function or indicative of an intermediate state into the evolution to decompensation and future heart failure. Further studies will be needed to determine if the detected CH detected in this model evolves to heart failure.

The comparison of the transcriptome of the ECs at 8 months post-PE with the same analysis performed (in our previous study) at the end of gestation, shows that most differentially expressed genes (DEGs) are different. For instance, at 8 months post-PE, we did not find clusters of genes directly involved in fibrosis and cardiac hypertrophy, nor a specific down regulation of genes involved in mitosis and cell division, which were strikingly enriched in preeclamptic pregnancies at delivery^18^. Instead, after 8 months, most upregulated DEGs were strongly associated with pathways related to inflammation (TNF-α signaling via NFκB, and interferon γ response) and cellular stress (hypoxia and apoptosis). In addition, a number of genes encoding proteins with anti-angiogenic properties were upregulated, such as *Adamts4* (fold change [FC] = 3.28, p = 0.009), *Akap12* (FC = 2.86, p = 0.024), or *Dcn* (FC = 2.86, p = 0.017)^44–46^. We also detected a significant upregulation of *Thbs1* (FC = 2.94, p = 0.0003) and *Selp* (FC = 1.70, p = 0.0087), encoding proteins promoting platelet aggregation as well as monocyte and neutrophil adhesion to ECs^47,48^.

*Shc1* is another gene significantly upregulated in ECs at 8 months post-PE (FC = 2.77, p = 0.009). *Shc1* encodes 3 main isoforms that differ in activities and subcellular location. All three are adapter proteins in signal transduction pathways; the longest (p66Shc) promotes oxidative stress and may be involved in regulating life span^49^. Homocysteine promotes EC dysfunction via upregulating p66shc expression by hypomethylation of specific CpG dinucleotides in the *Shc1* promoter^50^. Also, downregulation of p66shc increases endothelial NO synthase activity and improves endothelium-dependent vasorelaxation^51^. Ablation of p66shc protects against angiotensin II (Ang II)-induced cardiac hypertrophy and cell death^52^. In an atherosclerosis murine model, loss of p66shc protected vessels against oxidative stress damage^53^.

A hallmark of the ECs transcriptome analyzed 8 months post-PE is the strong upregulation of *Il*-6 (FC = 3.97, p = 0.015). System biology analysis showed that Il-6 has a central position (hub) in the network describing the physical and functional interactions among the proteins encoded by the DEGs in the ECs both at delivery (after PE) and 8 months post-PE. Moreover, Il-6 is involved in the pathogenesis of several CVDs. Clinical studies have established the role of Il-6 in the pathogenesis of atherosclerosis^54^, ischemic heart disease^55^, hypertension^56^, chronic heart failure and myocardial hypertrophy^55^, and several nephropathies including chronic kidney disease^57^. Il-6 induces significant physiological modifications, including activation and maintenance of low-grade systemic inflammation, disruption of EC homeostatic functions (impaired protection against reactive oxygen species [ROS]), EC dysfunction (reduced nitric oxide production, impaired relaxation, increased adhesion molecules, etc.), activation of monocytes, platelet aggregation, intimal proliferation, myocardial hypertrophy, and metabolic disturbance (leading to insulin resistance). Several inflammatory molecules, such as IL-1, lipopolysaccharide, TNF-α, and IL-4, stimulate the production of IL-6 in ECs. In turn, IL-6 promotes Ang II type 1 receptor gene expression and leads to Ang II-induced vasoconstriction and ROS production, which results in EC dysfunction^58^.

Severe PE has been associated with high circulating levels of cytokines including TNF-α, IL-8, IFN-γ and also IL-6, whereas normotensive pregnancy presents high level of the regulatory cytokine IL-10^59^. In women with PE, several plasmatic inflammatory markers showed altered expression, in particular IL-6/IL-10 ratio, 20 years after the disease^60^, which suggests a persistent imbalance between pro- and anti-inflammatory circulating molecules. In the STOX1 model at 8 months post-experimental PE, we observed mild alterations of the cytokine profile that nevertheless allowed us to identify combinations of cytokines with abnormal expression that could help propose a set of plasma markers to evaluate the inflammatory status of the patient and propose adequate follow-up. Surprisingly, IL-6 was a notable transcript with increased expression in ECs of mice with previous PE, but this did not translate into increased circulating levels of IL-6, which may contradict the literature^60^. A recent study^61^ examined C-reactive protein (CRP) level, IL-6, and intercellular adhesion molecule 1 in the plasma of patients with a history of hypertensive disorder of pregnancy, 17 years post-pregnancy, on average, and showed increased IL-6 level (by 34.4%) and CRP level (by 11.6%) as compared with controls. These increases were relatively moderate and based on the analysis of a large number of patients (2614 and 2490 women for CRP and IL-6, respectively, 10% of whom had an history of hypertensive disorder of pregnancy). Consistent with our findings, 2 articles showed that deficiency of IL-4 and Il-10 induced or aggravated PE symptoms in mice^62,63^. We found a decreased level of these 2 cytokines in endothelium and plasma in the long-term and so validates our findings but this should be strengthened with an increased number of animals since a limitation of our study is the number of mice analyzed in each group, suggesting that our data have to be taken as preliminary.

Several studies with mice models have reported altered endothelial function after PE. Consistently, in a mouse model of PE induced by sFLT1 overexpression, the repair capability of the endothelium after carotid injury was altered at 2.5 months post-partum^64^. Bytautiene and coworkers induced PE in mice via adenoviral overexpression of sFLT1 at 8 days of gestation and evaluated vascular function 6 to 8 months after delivery^12^. Animals with preeclamptic pregnancy and normal pregnancy did not differ in BP or vascular function. The authors concluded that late CVD risk in women with PE was due to preexisting risk factors. However, later on, the same team, using the same PE model, examined the plasma proteome 6 months after delivery and reported an important deregulation of proteins involved in the inflammatory response, organismal injury, hematologic and metabolic disease^13^. From this later study, the authors concluded that some of the long-term adverse outcomes associated with PE actually may be a consequence rather than a mere unmasking of an underlying predisposition. WT C57BL/6 female mice impregnated with C1qKO males (deficient in C1q) constitute another model of PE^65^. A recent study using this model showed the persistence of EC dysfunction in females at 2 months post-partum as well as hypertension and glomerular injury^15^. The authors also reported non-transient LV hypertrophy after PE associated with ventricular remodeling. However, administration of pravastatin during PE prevented the long-term effects on CV health in this model of late-onset PE.

All these studies, using different models, report results very similar to what we observed with the STOX1 model, in which we detected the 8 months’ post-partum persistence of LV hypertrophy, fibrosis, altered cardiac function, modified endothelial transcriptome and mildly altered cytokine profile. Thus, our results strengthen those obtained with other murine PE models and are consistent with the hypothesis that PE by itself can cause long-term adverse CVD alterations. To the best of our knowledge, our study is the first to systematically analyze the gene expression profiles of ECs of mice at 8 months post-PE (corresponding to approximately 20 human years^66^). Our study clearly establishes that 8 months after PE, the ECs display a transcriptome profile characteristic of an inflammatory condition. This transcriptomic analysis represents a first glance at the altered molecular mechanisms at work in the endothelium, which compromises CV health.

An important question is the origin of these sustained alterations. During PE, the placenta releases factors in the maternal circulation that adversely affect the vascular system independent of any preexisting risk. This impact remains imprinted in the vascular system and leads to vascular dysfunction with aging. Several hypotheses can be evoked. PE could alter the composition of different sub-populations of ECs such as EC precursors, an issue that could be studied by single-cell RNA sequencing approaches, for example. Alternatively, abnormal epigenetic markers (such as DNA methylation) could have been affixed at specific genomic regions, altering gene expression throughout cell generations (an example of this could be the hypomethylation of *SHC1* in ECs described above). Another possibility is the persistence after PE of an activated population of immune cells that could maintain a low-grade inflammation status, which with time will lead to EC activation and dysfunction. This issue will be the purpose of future studies. Finally, it could be suspected that foetal cells persist in the maternal organism long after pregnancy (in our case, these cells would overexpress STOX1, potentially leading to long term effects). The existence of such cells in mice is now clearly established^67^, but they are in limited number (66–420) in the lungs at the end of pregnancy. Therefore, we consider that it is doubtful that the phenotype observed 8 months after pregnancy is due to such persistent cells.

## Supplementary information

Supplementary Material and Methods + Supplementary Figures and their legends

Supplementary Table 1

Supplementary Table 2
