# Supplementary material for: Long-term cardiovascular disorders in the STOX1 mouse model of preeclampsia
Source: Sci Rep. 2019 Aug 15;9:11918. doi: 10.1038/s41598-019-48427-3 (PMC6695383; doi:10.1038/s41598-019-48427-3)

**Supplementary Information for**

**“Long term cardiovascular disorders in the STOX1 mouse model of preeclampsia”.**

Francisco Miralles^1^, Hélène Collinot^1^, Yasmine Boumerdassi^1^, Aurélien Ducat^1^, Angéline Duché^2^, Gilles Renault^3^, Carmen Marchiol^3^, Isabelle Lagoutte^3^, Céline Bertholle^4^, Muriel Andrieu^4^, Sébastien Jacques^2^, Céline Méhats^1^, Daniel Vaiman^1*^

^1^Institut Cochin, U1016 INSERM - UMR8104, CNRS – Université Paris Descartes, Team ‘From Gametes To Birth’ 24 rue du Faubourg St Jacques, 75014, Paris, France

^2^Institut Cochin, U1016 INSERM - UMR8104, CNRS – Université Paris Descartes, Genom’IC Platform, Bâtiment Gustave Roussy, 27 rue du faubourg Saint Jacques, 75014 Paris, France

^3^Institut Cochin, U1016 INSERM - UMR8104, CNRS – Université Paris Descartes, PIV Platform, 22 rue Méchain, 75014 Paris, France

^4^Institut Cochin, U1016 INSERM - UMR8104, CNRS – Université Paris Descartes, CYBIO Platform, 27 rue du Faubourg Saint Jacques 75014 Paris, France.

* corresponding author.

Tel 00 33 1 44412301

Fax 00 33 1 44412302

Email: [daniel.vaiman@inserm.fr](mailto:daniel.vaiman@inserm.fr)

**Ultrasonography**

Before pregnancy, at 3 and 6 months post-pregnancy, ultrasonography (US) was performed with a 40MHz VisualSonics VeVo 2100 apparatus (Toronto, Canada), as described^1^ for Doppler analysis and the collection of 59 different US parameters. At 6 months, US was performed with or without dobutamine injection. Dobutamine functions through a direct stimulation of [β1 receptors](https://en.wikipedia.org/wiki/Beta_receptor) of the [sympathetic nervous system](https://en.wikipedia.org/wiki/Sympathetic_nervous_system), and has positive inotrope actions (increasing heart contraction^2^), Dobutamine injection was used early to mimic cardiovascular stress in mice ^3^ In our experimental design, animals were anesthetized with isoflurane (3% concentration in air for induction, 1.5% for US imaging), attached in supine position to a heating pad and monitored for temperature, respiration and heart rate by using a dedicated system (Vevo imaging station, Visualsonics). The mouse thorax was depilated by using depilatory cream, applying US contact gel before positioning the US probe (MS550D on a Vevo 2100 US imaging system, Visualsonics). Heart dimensions (septum and posterior wall thickness, left ventricular [LV] internal diameter), were acquired in parasternal long axis view with M mode; a modified parasternal short axis view was used to assess pulmonary artery velocities, aortic flow, aorta diameter and left atrium [LA] diameter measured in suprasternal position. Mitral flow was assessed in apical incidence. After baseline acquisition, mice underwent stress echocardiography, measuring the same parameters 8 min after injection of 1 µg/g dobutamine intraperitoneally. All measurements were collected with Vevolab (Visualsonics), and LV mass was calculated by using M-mode dimensions. At 7-8 months post-pregnancy, blood pressure was measured by *in vivo* tail measures for 5 consecutive days as described ^4^.The mice were sacrificed at 8 months post-pregnancy under xylazine-ketamine anesthesia. Plasma and organs (heart, kidney, brains) were collected and kept for tissue analysis in paraformaldehyde (48h) before transfer in 70% Ethanol, or TriZol for RNA preparation.

**Histology**

Hearts were transferred to 70% ethanol after 2 days in paraformaldehyde 4%. Then, white and Masson trichrome-stained slides were prepared at the histology platform of Cochin Institute (HistIM). They were analyzed under light microscopy with 40-fold magnification. Ten slides from the 10 mice were analyzed blindly for fibrosis marks. The 5 slides from previously preeclamptic mice were all identified unambiguously, as were 3 slides from non-preeclamptic mice; the 2 remaining slides could not be assigned.

**Purification of cd31+ cells and transcriptome analyses of the heart and endothelial cells (ECs)**

ECs purified from skeletal muscles at 8 months post-pregnancy as described ^5^ from this location for 3 different reasons: 1) to allow a direct comparison of the transcriptome with our previous study published in 2016 showing transcriptome alterations of the same cells at the end of preeclamptic pregnancy; 2) because PE is a general endothelial disease, we wanted to analyze other than the most evident organs for cardiovascular disease (CVD), and for this, muscles are good candidates, especially because skeletal muscle is the organ with major microvascular mass ^6^, and hypertension is known to be associated with dysfunctional ECs of the skeletal muscle ^7^; and 3) technically speaking, it was easier to obtain a sufficient amount of purified ECs that are rarer in ageing mice than in young mice than for example from other tissues such as mesentery or aorta. Briefly, muscles from the forelegs, hind legs and back were dissected and minced with small sterile scissors in a sterile glass plate, trying to remove fat and connective tissues. The tissues were incubated 20 min at 37°C in 6 ml collagenase B (Roche #11088831001, 3 ml 1.5 U/ml)/DispaseII (Roche #04942078001, 3 ml 2.4 U/ml))/CaCl_2_ 5 mM, homogenized every 5 min. In total, 500 µl of SVF was added to stop the reaction and the mix was filtered on a 70-µM filter. The cell suspension was collected after centrifugation and transferred to mouse anti-CD31 pre-coated Dynabeads. After binding, the beads were rinsed 5 times in sterile washing buffer: phosphate buffered saline, Fetal Calf Serum [FCS] 1%, and 2 mM EDTA and collected in Trizol after the last rinse. The yields varied according to the mice, from 62 to 2013 ng, but in all the cases, the RNA Integrity Number [RIN] was 7.5 and the quantity large enough for microarray analysis. The RNA from the heart was also prepared by using classical TriZol procedures. The samples were analyzed by using Clariom S mouse microarrays170. The raw microarray hybridization results will be available in Array express (accession nos. E-MTAB-7357 and E-MTAB-7358), for heart and ECs, respectively. The hybridizations were performed at the genomic and transcriptomic platform of Cochin Institute, according to standardized procedures. Quality control, normalization and statistical analysis were performed by using Transcriptome Analysis Console (TAC 4.0) (ThermoFisher Scientific). The Robust Multi-Array Average (RMA) method was applied for background correction, quantile normalization, multi-array summarization and log2-transformation of raw expression values. Differentially expressed genes (DEGs) were identified by calculating the mean expression log2 fold change between sample groups (experimental preeclampsia [PE] versus control). Statistically significant DEGs were selected by using the Limma moderated *t* test. Finally, the Benjamini & Hochberg procedure was for multiple testing adjustment to control the false discovery rate (FDR).

**Multiplex simultaneous analysis of 33 cytokines and logistic regression analysis**

The 10 plasma samples were analyzed with the BioRad mouse chemokine panel 33-plex kit according to the manufacturer's instructions and read on Bio-rad Bioplex200 system. Each measure was made in duplicate. Standard curves were calculated from the standard samples and the values were fitted to a Logistic-5Pl curve, allowing to estimate the concentrations of the relevant samples.

**Bioinformatics analyses**

Over-representation analysis (ORA) of the DEGs was performed with the WebGestalt (<http://bioinfo.vanderbilt.edu/webgestalt>) bioinformatics resource ^8^. WebGestalt incorporates updated information from different public databases and provides a way to generate sensible biological information out of gene lists. The databases interrogated were Gene Ontology (GO), Kyoto Encyclopedia of Genes and Genomes (KEGG), WikiPathways and Hallmarks ^9^. The significance of the detected enrichments was calculated using the Benjamini & Hochberg multiple test adjustment. Ontologies and pathways with adjusted P < 0.05 were considered significant. The Hallmarks network presented in Figure 5 was constructed, visualized and analyzed by using the Cytoscape 3.6.0 software and its complementary applications (Cytoscape Apps) ^10^. In this network, the nodes represent DEGs and edges the relationship among genes (in that case the attribution to a given gene ontology or pathway). The fold change values (calculated as experimental PE versus control) of the DEGs was incorporated in the network to visualize up- or downregulation. In addition, we performed Gene Set Enrichment Analysis (GSEA) to determine whether predefined groups of genes (called Gene Sets) were asymmetrically distributed or not in a gene expression dataset. Each Gene Set is a group of correlated genes because they are coexpressed, involved in the same pathway, or colocalized in the same cellular compartment, etc. GSEA ranks all genes in an expression dataset, then calculates an enrichment score (ES) for each gene set, which reflects how often members of that gene set occur at the top or bottom of the ranked dataset (i.e., the most highly expressed genes or most under-expressed genes). The statistical significance of the ES is calculated by using a phenotypic-based permutation test to produce a null distribution for the ES. The ES for each set is normalized, generating a normalized enrichment score (NES), which takes into account the size of the gene sets and allows for comparing the analysis results across gene sets. Finally, an FDR is calculated to adjust for multiple hypothesis testing. The complete gene expression dataset issued from our microarray analysis was analyzed by using the Broad Institute GSEA software (<http://software.broadinstitute.org/gsea/index.jsp>). GeneSet “Hallmarks”, encompassing 50 non-redundant groups of genes involved in important cellular basic function, were searched. A GeneSet was considered significantly enriched with FDR < 0.25 for the NES (the default setting classically used for this type of analysis).

**Statistical analysis**

Student *t* test was used to compare 2 groups of mice. The US results were analyzed by ANOVA: for the measures at 0, 3 and 6 months, repeated-measures ANOVA was used, which allowed for estimating a significant effect of time in the variable measured and an interaction of this variation with the placental genotype during gestation (controls or overexpressing STOX1). For dobutamine treatment, the analysis was exclusively at 6 months and a double-way ANOVA allowed for estimating 1) the genotype effect, 2) the dobutamine effect, and 3) the interaction effect between the two factors. The test results are in Supplementary Table 1, and the effects of dobutamine at 6 months by placental genotype are as Figure 2. P <0.05 was considered statistically significant. Then we calculated Discriminant Function Coefficients, which led to the following equation for the 8 most discriminant cytokines found in mouse plasma: Discriminant Factor (DF) = 6.73 10^-3^ x [Cxcl13] -1.69 10^-2^ x [Cxcl16] - 1.39 10^-2^ x [Cxcl11] +2.90 10^-3^ x [Il-16] – 1.37 10^-2^ x[Il-10] + 9.75 10^-1^ x [Il-2] – 4.61 x [Il-4] - 3.23 10^-1^ x [Ccl1]. Discriminant analysis, ANOVAs and Student *t* tests were performed with the Statist’XL add-on of Microsoft Excel. The details of the discriminant analysis calculations are in Supplementary Figure 3.

**References**

1. Collinot H, Marchiol C, Lagoutte I, Lager F, Siauve N, Autret G, Balvay D, Renault G, Salomon LJ and Vaiman D. Preeclampsia induced by STOX1 overexpression in mice induces intrauterine growth restriction, abnormal ultrasonography and BOLD MRI signatures. *J Hypertens*. 2018;36:1399-1406.

2. Tuttle RR and Mills J. Dobutamine: development of a new catecholamine to selectively increase cardiac contractility. *Circ Res*. 1975;36:185-96.

3. Mor-Avi V, Korcarz C, Fentzke RC, Lin H, Leiden JM and Lang RM. Quantitative evaluation of left ventricular function in a TransgenicMouse model of dilated cardiomyopathy with 2-dimensional contrast echocardiography. *J Am Soc Echocardiogr*. 1999;12:209-14.

4. Doridot L, Passet B, Mehats C, Rigourd V, Barbaux S, Ducat A, Mondon F, Vilotte M, Castille J, Breuiller-Fouche M, Daniel N, le Provost F, Bauchet AL, Baudrie V, Hertig A, Buffat C, Simeoni U, Germain G, Vilotte JL and Vaiman D. Preeclampsia-like symptoms induced in mice by fetoplacental expression of STOX1 are reversed by aspirin treatment. *Hypertension*. 2013;61:662-8.

5. Ducat A, Doridot L, Calicchio R, Mehats C, Vilotte JL, Castille J, Barbaux S, Couderc B, Jacques S, Letourneur F, Buffat C, Le Grand F, Laissue P, Miralles F and Vaiman D. Endothelial cell dysfunction and cardiac hypertrophy in the STOX1 model of preeclampsia. *Sci Rep*. 2016;6:19196.

6. Wang MX, Murrell DF, Szabo C, Warren RF, Sarris M and Murrell GA. Nitric oxide in skeletal muscle: inhibition of nitric oxide synthase inhibits walking speed in rats. *Nitric Oxide*. 2001;5:219-32.

7. Payne GW. Effect of inflammation on the aging microcirculation: impact on skeletal muscle blood flow control. *Microcirculation*. 2006;13:343-52.

8. Zhang B, Kirov S and Snoddy J. WebGestalt: an integrated system for exploring gene sets in various biological contexts. *Nucleic Acids Res*. 2005;33:W741-8.

9. Subramanian A, Tamayo P, Mootha VK, Mukherjee S, Ebert BL, Gillette MA, Paulovich A, Pomeroy SL, Golub TR, Lander ES and Mesirov JP. Gene set enrichment analysis: a knowledge-based approach for interpreting genome-wide expression profiles. *Proc Natl Acad Sci U S A*. 2005;102:15545-50.

10. Shannon P, Markiel A, Ozier O, Baliga NS, Wang JT, Ramage D, Amin N, Schwikowski B and Ideker T. Cytoscape: a software environment for integrated models of biomolecular interaction networks. *Genome Res*. 2003;13:2498-504.

**Supplentary Figures Legends**

**Supplementary Figure 1**

Time Chart of the present experimental study and presentation of previous data on the STOX1 mouse model.

**Supplementary Figure 2**

Absolute values of the Normalized Enrichment Scores (NES) for the clusters composed of up-regulated genes (in red) and down-regulated genes in blue. The FDR p-value were <0.023 for the induced genesets, from 0.16 to 0.27 for the down-regulated genesets, indicating that in the endothelial cells, the genes are up-regulated in a concerted fashion, while this is totally untrue for down-regulated genes.

**Supplementary Figure 3**

Whiskers plots of the 8 most discriminant cytokines found in the mouse plasma samples. Their combination in a discriminant regression allowed to separate the two mouse groups (See Figure 6).

**Supplementary Figure 4**

Presentation of the 33 cytokines analyzed in the BioPlex kit in the plasma, in regard with their relative level of RNA in the endothelial cells (Induction ratio, and its logarithm). The calculated coefficients of the discriminant equation are given. Multiplying each of them with the value given for the corresponding cytokine in a mouse results in a value ranging from -8.36 to -9.00 in control mice and from -3.59 to -6.61 in mice that had a preeclampsia (figure 6B).


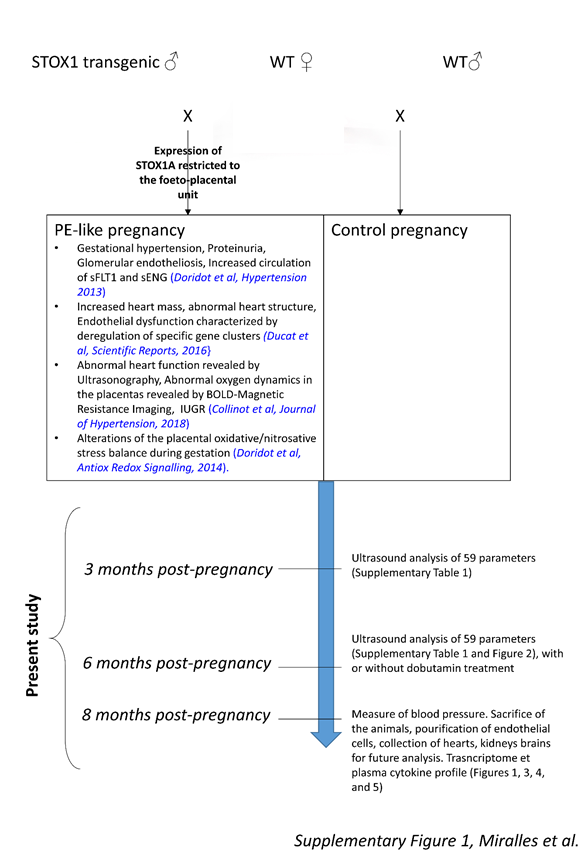

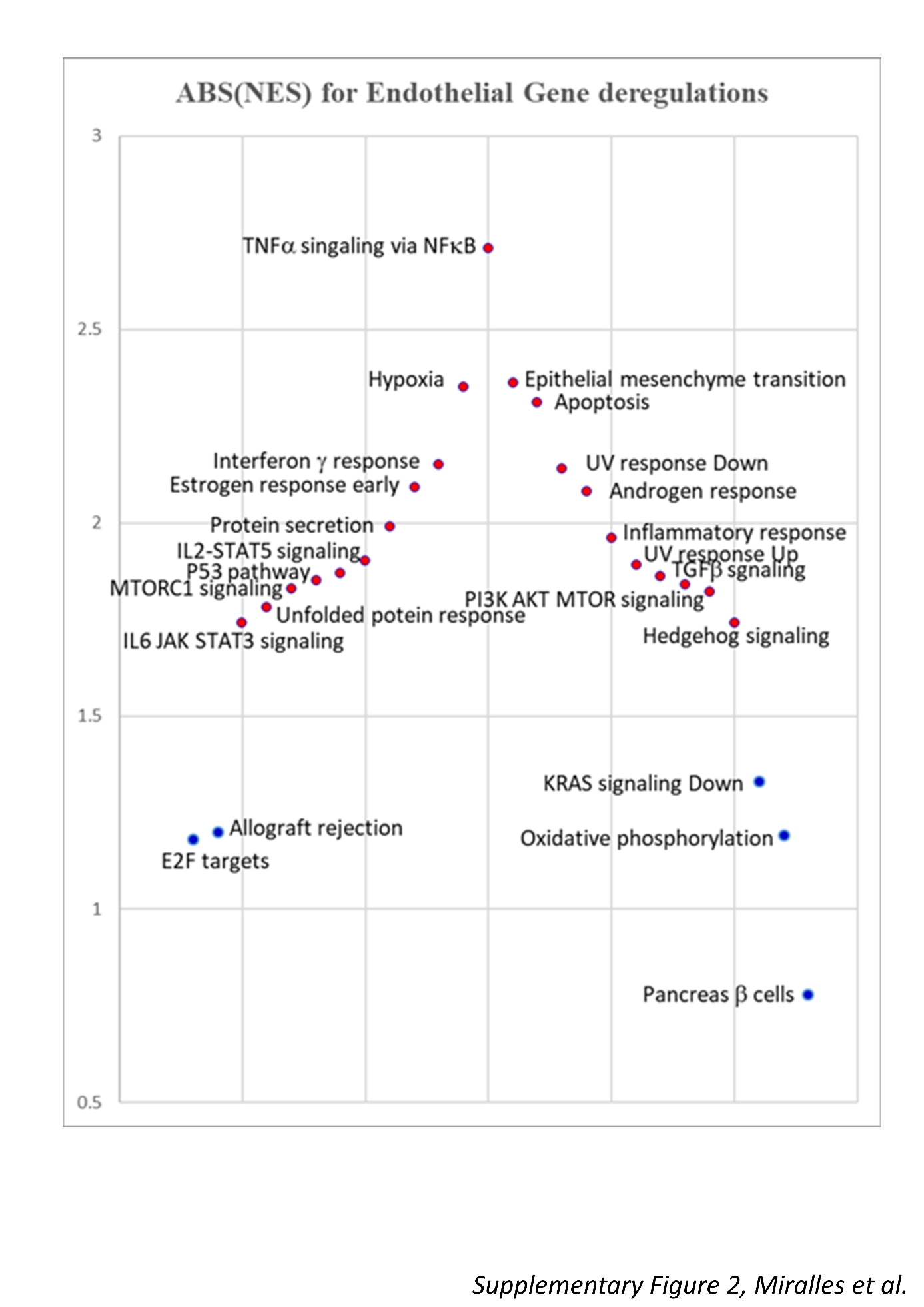


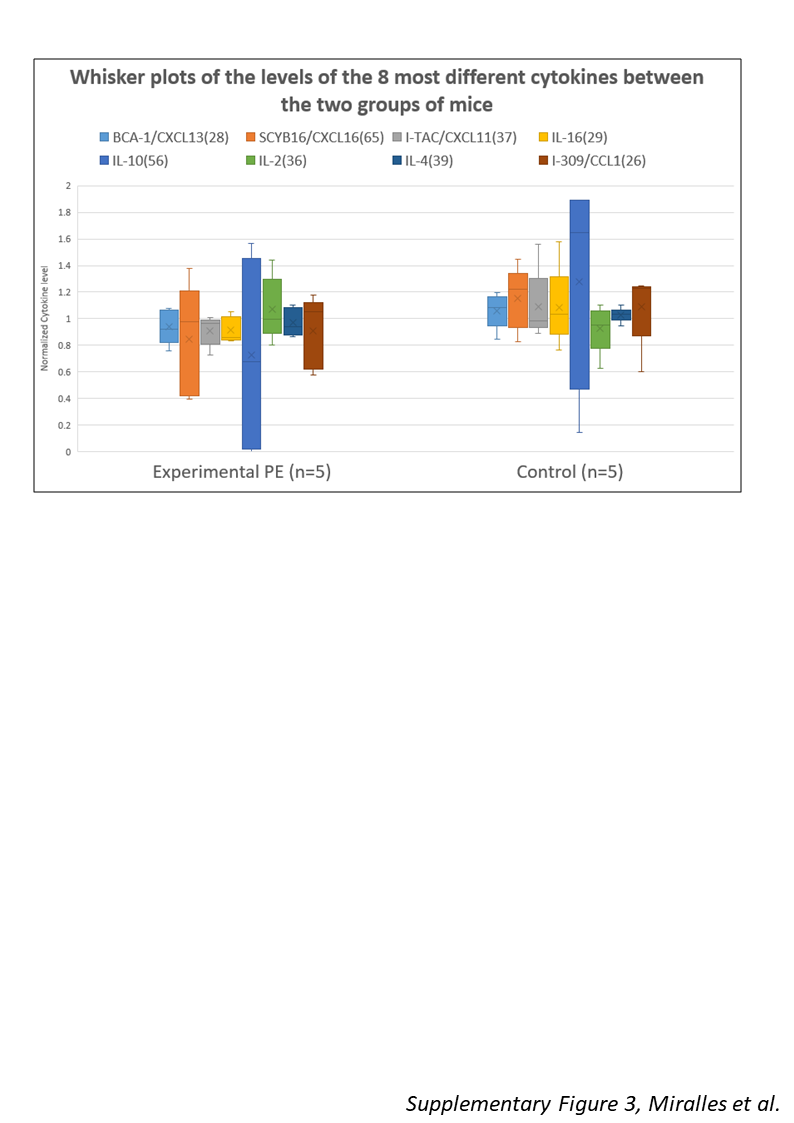

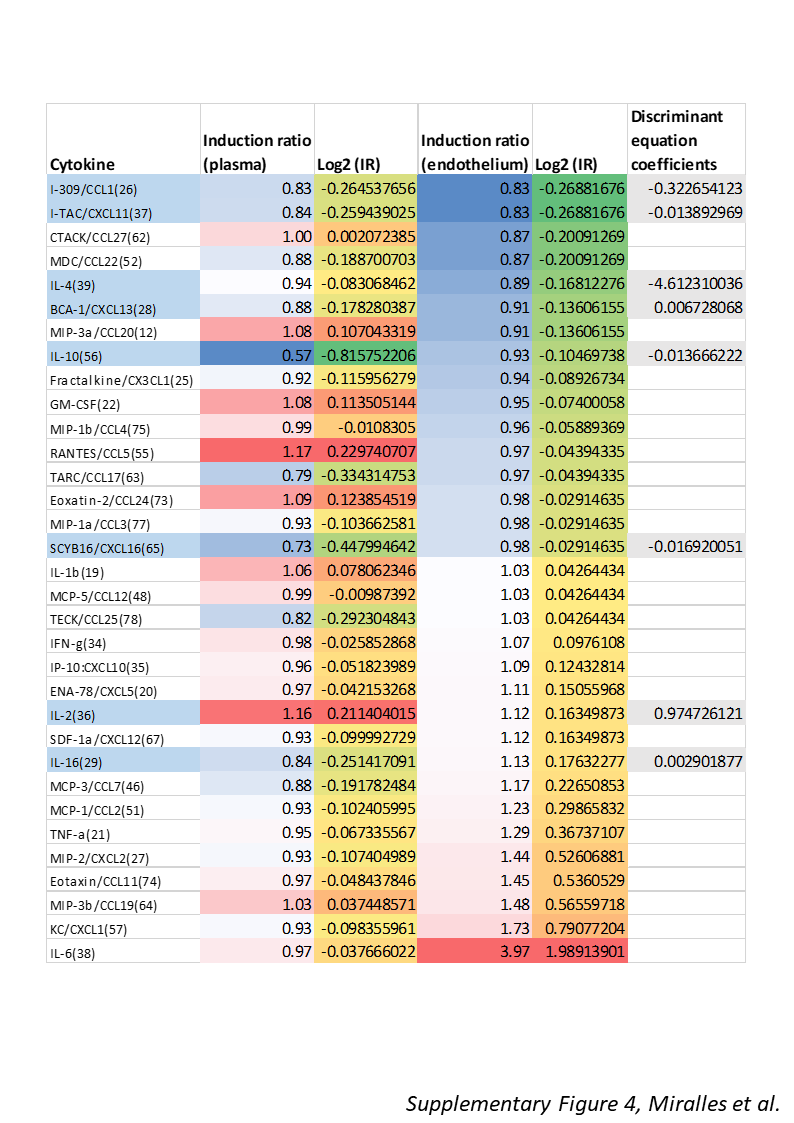

Supplement: Supplementary file 1 — Supplementary Material and Methods + Supplementary Figures and their legends [file 41598_2019_48427_MOESM1_ESM.docx]
